# Investigating the Influence of a Tooth Absence on Facial Bone Growth Using a Porcine Model

**DOI:** 10.3390/ijms252312509

**Published:** 2024-11-21

**Authors:** Dominika Szkopek, Piotr Wychowański, Kamil Zaworski, Blanka Seklecka, Rafał Starzyński, Paweł Lipiński, Kateryna Pierzynowska, Stefan G. Pierzynowski, Janine Donaldson, Łukasz Paczewski, Jarosław Woliński

**Affiliations:** 1 Laboratory of Large Animal Models, The Kielanowski Institute of Animal Physiology and Nutrition, Polish Academy of Sciences, Instytucka 3, 05-110 Jabłonna, Poland; d.szkopek@ifzz.pl; 2 Oral Surgery and Implantology Unit, Division of Oral Surgery and Implantology, Department of Head and Neck, Institute of Clinical Dentistry, Fondazione Policlinico Universitario A. Gemelli IRCCS, Universita Cattolica del Sacro Coure, 00168 Rome, Italy; 3 Department of Animal Physiology, The Kielanowski Institute of Animal Physiology and Nutrition, Polish Academy of Sciences, Instytucka 3, 05-110 Jabłonna, Poland; k.zaworski@ifzz.pl (K.Z.); or kateryna.pierzynowska@biol.lu.se (K.P.); 4 Phase Research Team, Adamed Pharma S.A., 05-152 Pieńków, Poland; blankaseklecka@gmail.com; 5 Department of Molecular Biology, Institute of Generics and Animal Biotechnology PAS, 05-552 Jastrzębiec, Poland; r.starzynski@igbzpan.pl (R.S.); p.lipinski@igbzpan.pl (P.L.); 6 Department of Biology, Lund University, 221 48 Lund, Sweden; stefan.g.pierzynowski@gmail.com; 7 Anara AB, 231 32 Trelleborg, Sweden; janine.donaldson@wits.ac.za; 8 Department of Medical Biology, Witold Chodźka Institute of Rural Medicine, 20-090 Lublin, Poland; 9 School of Physiology, Faculty of Health Sciences, University of the Witwatersrand, Parktown, Johannesburg 2193, South Africa; 10 E-Medical Center Profivet sp. z o.o. sp. k, 05-190 Nasielsk, Poland; paczewski@gmail.com

**Keywords:** bone growth development disorders, anodontia, animal model, tooth buds, growth factors

## Abstract

With the current state of knowledge regarding disorders of facial bone development, including anodontia, the development of a suitable animal model for preclinical studies is essential. The agenesis of dental buds occurs in about 25% of the human population. Prospects for treatment include the use of growth factors, stem cells, and bioengineering. This study aimed to investigate the influence of a tooth absence on facial bone growth, develop a technique for the application of growth factors to the developing bone, and analyze the comparative effect of the application of selected active proteins on the growth of the maxilla and mandible. Piglets underwent germectomy, followed by computed tomography and X-ray; morphometric and histological analyses of the bones were performed, blood bone morphogenetic protein 2 and platelet-derived growth factor concentrations were determined, and the transcriptomic profile of the dentate ligament was analyzed using DNA microarrays. It was not possible to identify the most effective growth factor application algorithm for achieving normal jaw development. Normal mandibular bone structure and oral mucosa structure were observed in the germectomy groups with growth factor augmentation. The average height of the mandibular alveolar part in the area of the removed dental buds was significantly lower compared with that of the inoperable side, 3 months after surgery. However, no significant differences were found in the serum concentrations of BMP-2 and PDGF between groups. The animal model of bone development disorders (including anodontia) developed in the current study and the scheme for evaluating the efficacy and safety of the application of replacement therapy for craniofacial malformations are important in the development of the discipline and represent an important contribution to the introduction of treatment methods.

## 1. Introduction

Tooth loss caused by physical trauma, tooth decay, genetic defects, and aging are prevalent health problems [1]. The lack of tooth buds has numerous complications. In addition to reduced chewing efficiency due to the presence of free space in the dental arch, the teeth begin to migrate, leaning toward the gap, and teeth of the opposite arch, through the so-called Godon’s symptom, show extrusion from the alveoli, which may cause premature occlusal contacts and obstacles during jaw movements and subsequent disorders in the dental system [2,3,4]. Tooth agenesis is also associated with disorders in the development of jaw bones. Recent studies have shown that the facial dimensions of patients with agenesia of at least one tooth are on average 0.56–0.59% smaller than in the corresponding control group. Changes in skull bone dimensions are correlated with the number of missing teeth [5]. It is estimated that with the agenesia of one tooth, the dimensions of human jaws are reduced by 0.5 to 1 mm [6]. The influence of dental buds on the surrounding bone is still being studied, but it is known that they are the source of numerous growth factors regulating the metabolism of the surrounding tissue [7]. The formation of tooth buds is the result of epithelial–mesenchymal interactions. Different stages of organogenesis involve different growth factors, mainly from the FGF, EGF, TGF-β, BMP, and PDGF families [8,9].

The process of teeth eruption, through coordinated processes of resorption and bone tissue positioning, has an impact on alveolar bone development. It has been experimentally proven that the compound bellows are essential for the initiation of the production of the eruption canal for an erupting tooth [10]. Bone tissue resorption is intensified from the crown side of the compound and this process is reflected in the increased expression of genes such as RANKL, CSF-1, and MCP-1 [11,12]. The expression of factors such as osteoprotegrin (OPG) or secreted frizzled-related protein-1 (SFRP-1), responsible for the inhibition of osteoclastogenesis is reduced [7,12,13]. At the apex side, the bone tissue is simultaneously adsorbed, accompanied by increased expression of, among others, bone morphogenetic proteins (BMP-2, BMP-3, BMP-4, and BMP-7) [1,14,15,16]. The development and eruption of teeth are mechanisms in which the regulation of the expression of individual genes, both in time and spatial position, determines proper teeth formation, which, in turn, affects alveolar bone [14]. The cells of the compound follicle produce significant amounts of PTHrP (parathormone-related peptide), which, by acting locally on mesenchymal progenitor cells, determines their differentiation into root cement cells, osteoclasts, osteoblasts, or ligament cells. Communication via the autocrine/paracrine ligand pathway, i.e., PTHrP, is essential for the development of the tooth, the process of ejection, and the coordinated development of periodontal tissues, including alveolar bone [17,18]. The treatment of patients affected by the agenesis of tooth buds depends largely on the severity of the observed disorders and the number of missing teeth. During the developmental period, the use of permanent prosthetic restorations or implantoprosthetic solutions is mostly avoided due to the ongoing process of growth of the cranial skeleton. Treatment of patients with hypodontia, oligodontia, and facial bone development disorders requires the collaboration and coordination of the actions of doctors of many specialties: orthodontics, dental surgery, prosthetics, and conservative dentistry. The aim of the treatment is to ensure correct chewing function and improvement of aesthetics as the congenital lack of teeth can translate into psychosocial problems and a subjective lower quality of life [19,20]. Optimal effects are achieved by combining orthodontic preparation with implantoprosthetic treatment; however, the lack of a stimulating effect of tooth compounds on the surrounding alveolar bone makes it necessary to perform additional regeneration procedures, augmenting the bone tissue dimension [21]. An alternative to dental implants is the autotransplantation of teeth, which provides a physiological effect on the alveolar bone and follows the pattern of the normal growth process [22,23]. Future treatment methods may involve the use of growth factors, stem cells, and bioengineering. These options are still in the research phase, but progress in these branches of medicine may bring about new treatment options for patients suffering not only from tooth loss due to periodontal disease, caries, or trauma, but also for those suffering from congenital lack of tooth compounds [24,25,26].

New therapeutic perspectives cannot be introduced into clinical reality without first being tested under experimental conditions. There is an important need to develop animal hypodontia models that allow for repeated developmental research on therapeutic options. In this paper, we present a porcine animal model, reproducing the key disorders observed in the agenesis of dental buds, which may provide a basis for further work in this field.

Considering the above, we hypothesized that the surgical removal of the dental buds and the growth factors they contain would have an effect on the growth and development of the facial bones of the pig, a suitable animal model for this type of study. In addition, this study aimed to develop a technique for the application of growth factors to the developing bone and analyze the comparative effects of the application of selected active proteins on the growth of the maxilla and mandible.

## 2. Results

### 2.1. Cone Beam Computed Tomography and Manual Measurements on the Mandible Bones

Mandibular measurements showed no statistically significant differences in bone dimensions in terms of the length and width of the mandibular shaft and the dimensions of the mandibular branches between groups. The average height of the mandibular alveolar part in the area of the removed dental buds was significantly lower (*p* = 0.0072) compared with that of the inoperable side, 3 months after surgery (2.66 ± 0.12 cm and 3.35 ± 0.11 cm, respectively). The external and internal mandibular measurement results are presented in Table 1 and Table 2, respectively. All of the observed changes, according to the available literature, cause significant disturbances in the process of craniofacial bone development. The studies performed did not confirm the effectiveness of the proposed therapy in the form of injections of growth factors into the wound of the locus after the eruption of the dental cords since no significant effects of the injections on the process of facial bone growth were demonstrated. Consideration should be given to administering the tested factors for a longer period of time or in increased doses. The normal histological structure of the tested bones and osteointegration of the implanted fillers—collagen and TCP—with the recipient tissues were also demonstrated.

### 2.2. Analysis of Platelet-Derived Growth Factor (PDGF-bb) and Bone Morphogenic Protein 2 (BMP-2) Concentration in Piglet Serum

No significant differences in PDGF-bb and BMP-2 levels in the peripheral blood of piglets were observed between groups (Table 3 and Table 4). PDGF-bb and BMP-2 levels were shown to be constant on the three consecutive days tested (before and at 30 and 60 min after local injection of the test substances). Administration of PDGF (Group 3, by injection) and BMP-2 (Group 2, by injection) did not affect the peripheral concentration of the tested factor.

### 2.3. Microarray Analysis on Mandibular Tissue Samples

**Figure 1:**
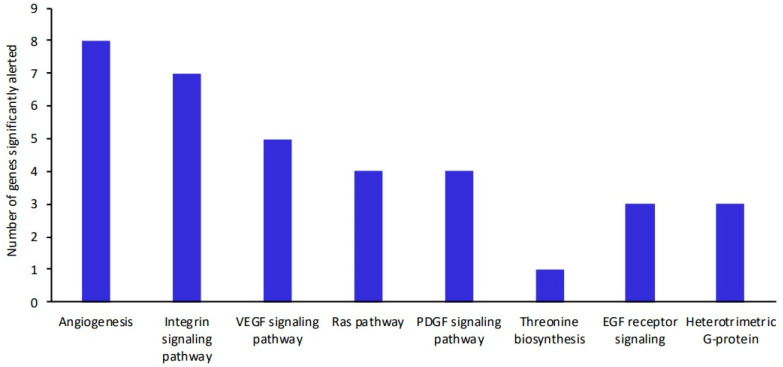
Graphical summary of the signaling pathways occurring in the tooth buds of the piglets.

**Figure 2:**
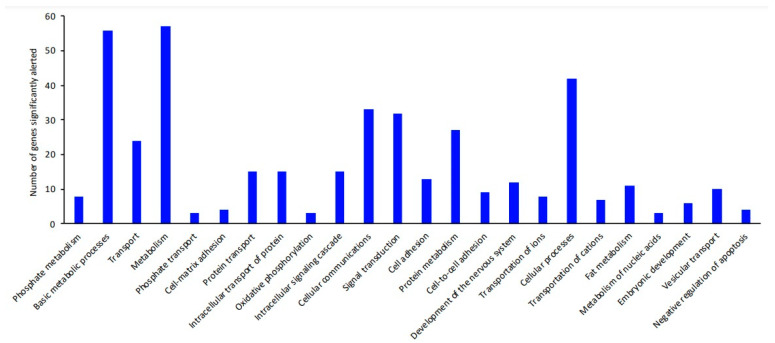
Graphical summary of the biological processes occurring in the tooth buds of the piglets.

The biological processes and signaling pathways occurring in the dentine ligament are presented in Table 5 and Table 6 and Figure 1 and Figure 2. The most significant statistical differences were obtained in the integrin signaling pathway (*p* = 2.09 × 10^−5^), and there were also significant changes in the signaling pathway for PDGF (platelet-derived growth factor) (*p* = 6.06 × 10^−3^). The most significantly affected processes were the processes of intercellular communication (*p* = 1.04 × 10^−2^), signal transduction (*p* = 1.11 × 10^−2^), cell adhesion (*p* = 1.16 × 10^−2^), and protein metabolism (*p* = 1.42 × 10^−2^), (see Table 6 and Figure 2 for a full list of biological processes). 

### 2.4. Histological Examination

The erupted teeth in the control group had a normal structure and consisted of a heterogenic structure composed of crown, dentin, root, enamel, connective tissue, and alveolar bone. The analyzed preparations showed the normal structure of the dental canals, pulp, enamel, amyloblasts, odontoblasts, and blood vessels. Normal mandibular bone structure and oral mucosa structure were observed in the germectomy groups with growth factor augmentation.

## 3. Discussion

Experimental research on animal models aims to reproduce, as closely as possible, the conditions encountered in clinical situations, to investigate the etiology of diseases, as well as imitate physiological processes of growth, surgical conditions, and tissue healing.

The development of treatment methods based on biologically active molecules and stem cells and advances in bioengineering determine the growing need for research based not only on reductionist in vitro studies, which, despite their advantages, cannot faithfully reproduce complex processes taking place in the living organism. Therefore, it is necessary to conduct research using animal models. In the area of maxillofacial and dental surgery, depending on the purpose of the study and the procedures performed, various animal models have been proposed: among others, small rodents like mice and rats or larger mammals like rabbits, dogs, and pigs [27,28].

The pig is used as a model animal in many different branches of experimental medicine due to the numerous similarities of the pig to the human body in terms of anatomy, physiology, metabolism, and cell transformation pathways [28,29]. Considering the purpose of the model described in this paper, the correlations in the structure and metabolism of pig bone tissue, which shows a greater degree of convergence with human bone structure than smaller animals, such as rats or rabbits, are particularly important [30]. Based on the critical size model of the bone defect, the rate of regeneration of pig bone tissue (1.2–1.5 µm/d) is similar to that of humans (1.0–1.5 µm/d) [31,32]. Bone tissue morphology, regeneration capacity, and remodeling rate, as well as mineral content and sponge bone architecture, are among the beneficial features for the selection of the pig as a model for research on bone development disorders. According to the literature, the greatest degree of similarity between the structural parameters and physiology of bone tissue is observed in dogs; however, using them in biomedical research encounters ethical resistance [30,33].

Due to the size of the pigs, the performance of the surgical procedure in the early days of life is not a technical problem. It is possible to use standard instruments used in dental and maxillofacial surgery. Also, additional materials such as drills, barrier membranes, dental implants, or fastening screws do not need to be subjected to special modifications.

It is worth considering the possibility of developing a model based on miniature pigs due to the reduction in breeding costs due to the limited size of the animals and lower nutritional demands. According to the available literature, despite significant convergence with larger pig varieties, the physiology of miniature pigs may show a greater degree of similarity to human bone tissue in terms of structure and rate of remodeling [32,34]. Because both the structure of teeth and periodontium show a high degree of similarity to human tissue, miniature pigs are often used in periodontology and implantology studies [27,28]. The smaller dimensions of the piglets’ skulls may, in the surgically induced model of hypodontia, be of increased technical difficulty despite the potential advantages described above.

As the etiology of innate agenesis of dental compounds is not fully understood and since the genetic background of co-hypodontic syndromes shows great diversity, the development of a unified model to work on growth disorders of the facial skeleton by producing transgenic strains of pigs seems to be currently out of reach. Hypodontia, which is the most common developmental defect of the craniofacial skeleton in humans, is defined by the absence of one or more teeth. Depending on the number of missing teeth, the condition can be subdivided into hypodontia where less than six teeth are missing; oligodontia where more than six teeth are missing; or anodontia where there is a complete lack of teeth. The agenesis of dental buds occurs in about 25% of the human population. The lack of formation of the third molars is most commonly observed (22.63% of the population on average), followed by the lack of the second mandibular premolars (2.91–3.22%) and lateral incisors in the jaw (1.55–1.78%) [35,36,37]. Additionally, third molar agenesis is becoming more common, generation after generation [37]. Excluding the third molars, the lack of the second mandibular premolars and lateral incisors accounts for 85% of hypodontic cases. Hypodontia is more common in the permanent dentition (1–13%) compared with the primary dentition (0.5–2.4%) [38], but there is also a correlation between the agenesis of deciduous teeth and their permanent successors: in the absence of a deciduous tooth, the risk of not having a permanent tooth formed at the corresponding point in the dental arch significantly increases [39,40,41].

According to a meta-analysis, agenesia (not counting third molars) ranges from 1.6 to 6.9%, depending on the population studied [38,42]. The frequency of occurrence of hypodontia varies geographically [43]. It is more frequent in East Asia and Europe but less frequent in Western Asia and North America [43,44]. Hypodontia may occur as an isolated disorder in non-syndromic form, and in syndromic form, as one of the symptoms of genetic syndromes such as ectodermal dysplasia, clavicle-cranial dysplasia, or Down’s syndrome [45,46,47].

The reconstruction of the pathological state of the lack of tooth compounds and the observed consequences for the surrounding alveolar bone allows for studies to be carried out the results of which can be translated to clinical conditions for both syndromic and non-syndromic forms of hypodontia. So far, no animal model has been described that imitates bone growth disorders due to a congenital lack of tooth compounds.

The animal model presented in this paper for the study of developmental disorders of the facial bones (growth and maturation), in the course of dental compound agitation, is a feasible option for most research facilities as it allows for achieving a satisfactory degree of efficiency and repeatability of germectomy in several-day-old piglets in a limited period of time. The risk of postoperative complications is difficult to assess due to the limited number of cases described; however, considering that all animals in the current study had no postoperative complications, it can be assumed that the probability of adverse events is limited. The piglet’s further life and acceptance by the mother and the group were not adversely affected. Significant inhibition of alveolar growth in animals deprived of dental compounds on the second day of life may be a model for research on the prevention of bone development disorders in the case of hypodontia induced through surgical methods (bone augmentation) or that of orthopedic disorders (osteodistriction) or those based on growth factors or stem cells. This model can also be used to study the upcoming bioengineering solutions to reconstruct the function of both teeth and their compounds during the developmental period and their relationship with adjacent tissues such as alveolar bone and periodontal structure.

In accordance with the guidelines (depending on the category), all new therapies and medical devices introduced to humans require preclinical studies on animals. The developed animal model of bone development disorders (including anodontia), as well as a scheme for evaluating the efficacy and safety of using replacement therapy for craniofacial malformations, is important in the development of the discipline and is an important contribution to the introduction of treatment methods.

As mentioned above, tooth loss can be caused by physical trauma, decay, genetic defects (hypodontia), and aging, so our results should be interpreted with caution as our study undoubtedly has its limitations. The main limitation is the difference between the proposed porcine model and genetically based diseases such as anodontia (surgical removal of tooth buds in the early postnatal period vs. genetic basis). The second limitation may be the age of the animals used in the study since, usually, genetic defects such as anodontia manifest themselves in adults. Therefore, surgical removal of tooth buds in the early postnatal period seems to be the only option to assess the process of bone growth and maturation, which, at the same time, does not allow for the assessment of anodontia of permanent teeth.

Finally, considering the results of our study and many years of practice in developing large animal models, it must be said that there are no perfect experimental animal models. In fact, each model only slightly mimics the clinical condition of a given disease entity and requires further work and improvement. The main known models (ferrets, minipigs, mice, shrews, reptiles, and fish) of tooth number abnormalities have provided us with a wide reference base for genetic abnormalities underlying anodontia and other abnormalities of tooth number. Now we know that congenital tooth agenesis is typically an isolated anomaly present in otherwise normally developed individuals, but it has also been associated with >150 syndromic conditions; thus, no model reflects human tooth agenesis in its entirety, especially when investigating the “healthy hypo/anodontia”. One should consider that despite constant investigation into the genetic background of anodontia and bone growth disorders, the scientific community still lacks functional studies, and one such functional study is described in our manuscript.

## 4. Materials and Methods

### 4.1. Animals and Surgical Procedure

The protocol for this study was approved by the II Local Ethical Committee for Animal Testing in Warsaw, Poland (resolution no. WAW2_29/2016 from 6 July 2016). The study was carried out on 42 pigs (large white, 2 days old, female and male) from eight litters born on time, without complications. The breakdown of the experimental groups is shown in Table 1. Pigs underwent germectomy surgery on their 2nd day of life. The pigs were premedicated with a mixture of midazolam and methadone, intramuscularly. Preoxygenation was initiated during induction with isoflurane at a concentration of 5%. Surgery was performed under general inhalation anesthesia, with isoflurane at a concentration of 1.5–2% and oxygen flow at 1–1.5 L/min and saturated local anesthesia using 1.8 mL cartridges containing 40 mg of artemisin (artemisin hydrochloride) and 0.01 mg of adrenaline (adrenaline tartras) in 1 mL solution. Within the atrium of the oral cavity, in the area of the molars, a cut was made at the top of the alveolar process and a vertical release cut was made on the mesial side and a triangular mucosal-epidermal lobe was removed. Using a rubella drill on the turbine handpiece, with physiological saline cooling, the compact bone plate covering the dental plexus was removed. The compounds of the third and fourth premolar milk teeth on one side were removed. After smoothing the acute bone edges and cleaning the alveoli, the wound was sutured closed using 4-0 resorbable sutures (polyglycolid acid, Safil, Braun, Germany). During the procedure, an intramuscular antibiotic (at a dose of 8 mg of benzylpenicillin procaine and 10 mg of dihydrostreptomycin sulfate, Pen-strep, Scanvet, Poland) and analgesic drug (tolfenamic acid at dose 2 mg/kg, Tolfine, Vetoquinol, Poland) were administered intramuscularly. During the postoperative period, the pigs were given the same antibiotic and analgesic daily. The piglets were housed with their biological mother and the rest of their litter for 3 months. The postoperative period was without complications. Pigs were kept according to the standard breeding protocol under periodic veterinary care. Growth factors were administered to the animals as shown in Table 7. At three months post-surgery, the pigs were euthanased with pentobarbital sodium at a dose of 140 mg/kg, and mandibular tissues and blood were collected for further analyses.

### 4.2. Cone Beam Computed Tomography and Manual Measurements of the Mandible Bones

To evaluate changes in the dimensions of the mandible bones, measurements on CBCT (cone beam computed tomography) scans and manual measurements on the mandible bones after euthanasia of the pigs were performed.

Mandibular bones were extracted after euthanasia and cleaned of soft tissues with a raspator. Manual electronic caliper measurements were taken by the same researcher according to established reference points and lines. Overall dimensions of the mandible bones, mandibular branch, and mandibular shaft were measured, as well as the height and width of the alveolar process at 4 measurement points, covering the area from the first to the third milky premolars. Measurements of the dimensions of the alveolar process on the side free of dental buds were performed at the corresponding measurement points determined by the distance from the line connecting the most distal points of the condylar process and the mandible angle, in a line parallel to the lower edge of the mandible shaft.

**Figure 3:**
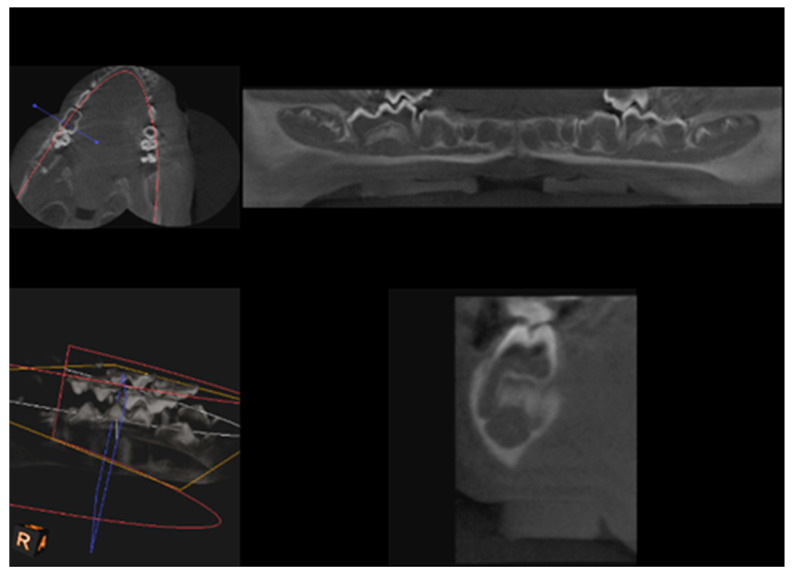
An example of a CT scan image of a piglet’s mandible before surgery to remove the dental setae.

**Figure 4:**
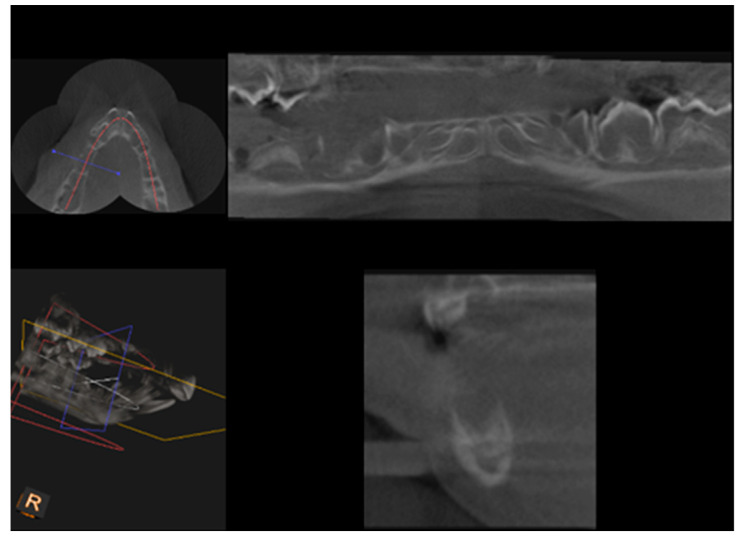
An image after removal of the dental setae together with the bellows.

**Figure 5:**
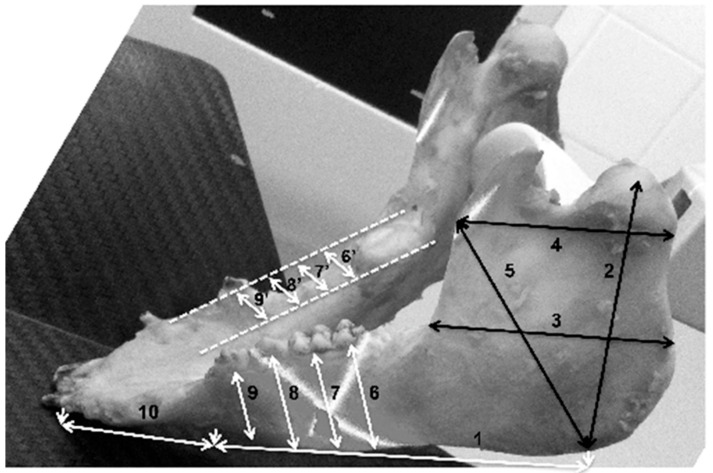
Diagram of external measurements of the mandible of piglets—left and right sides.

**Figure 6:**
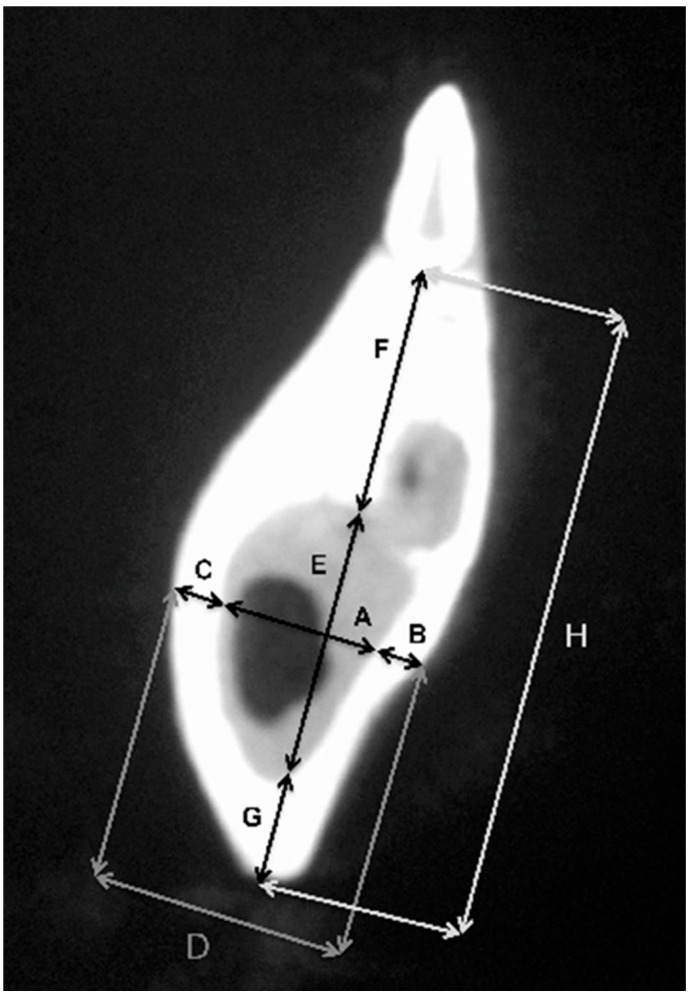
Diagram of internal measurements of the mandible of piglets—left and right sides.

CBCT examination, with a Kodak 3000C 3D system (Kodak Dental Systems, Atlanta, GA, USA), together with Dental Imaging Software ver. 6.12.32 (Kodak Dental Systems, Atlanta, GA, USA), was performed before and immediately after germectomy and then again on the extracted jaws of the piglets after euthanasia with a voxel size of 160 μm, a 0.16 mm space between slices and a field of view of 13 × 10 cm, 90 kV and 4 mA. CT sections were used to assess the width and height of the bone, the thickness of the base plate, the tongue and vestibular side, the dimensions of the marrow cavities, and the height of the mandibular alveolar part. Four measurement planes were determined in the area from the first to the third milky premolars on the dentition side, and then, similarly to the manual measurements, measurement planes were determined for the operated side at the same distance from the line connecting the most distal points of the condylar process and the mandibular angle, in a line parallel to the lower edge of the mandibular shaft. Figure 3 and Figure 4 show an example of a CT scan image of a piglet’s mandible before (Figure 3) and after surgery (Figure 4) and Figure 5 and Figure 6 are diagrams of the external (Figure 5) and internal measurements (Figure 6) of the mandible of the piglets.

### 4.3. Bone Morphogenetic Protein 2 (BMP-2) and Platelet-Derived Growth Factor (PDGF) Levels

Concentrations of BMP-2 and PDGF in the peripheral blood of piglets were determined before growth factor administration, thirty minutes after growth factor administration, and one hour after growth factor administration, for three days after treatment. Blood samples (2 mL) were obtained from the external jugular vein and collected into serum separator tubes. The blood was then centrifuged at 4700 RPM for 10 min and the serum was frozen at −80 until further analyses. Platelet-derived growth factor (PDGF-bb) was determined in pig serum using a Porcine PDGF-BB ELISA Kit (ThermoFisher Scientific, Waltham, MA, USA) according to the manufacturer’s protocol. Bone morphogenic protein 2—BMP-2 concentrations in pig serum were determined using a BMP2 ELISA Kit (ThermoFisher Scientific, MA, USA) according to the manufacturer’s protocol. The absorbance was measured using a spectrophotometer with a microplate reader (Multiskan Sky, ThermoFisher Scientific, MA, USA).

### 4.4. Microarray Analysis

Mandibular tissue samples were homogenized using TissueLyser LT (Qiagen, Germantown, MD, USA). Total RNA from homogenized tissue samples was isolated using an Rneasy Mini Kit (Qiagen, MD, USA). DNAse I digestion was included as part of the isolation protocol in order to ensure the sample was not contaminated with DNA. This was performed using an RNase-Free DNase Set (Qiagen, USA). RNA was quantified using a NanoDrop (NanoDrop Technologies, Wilmington, DE, USA). Final RNA quality and integrity were analyzed using an Agilent 2100 Bioanalyzer (Santa Clara, CA, USA) and an RNA 6000 Nano Kit (Agilent, Waldbronn, Germany). Only RNA samples with a relative integrity number (RIN) ≥ 7.8 were considered optimal and included in further analysis.

The gene expression profile was evaluated using an Agilent platform, including Agilent-028279 SurePrint G3 Rat GE 8x60K Microarray (Agilent Technologies, Santa Clara, CA, USA) and Agilent Technologies Reagent Set. All procedures were run according to the manufacturer’s protocols. An internal control, from the RNA Spike-In Kit (Agilent Technologies, USA) was added to all the samples, and amplification and labeling of target DNA were performed using a Low Input Quick Amp Labeling Kit (Agilent Technologies, CA, USA) in order to obtain complementary RNA (cRNA). Fragmentation and hybridization of cRNA were performed using a Gene Expression Hybridization Kit, and following hybridization, slides were washed using the Gene Expression Wash Buffer Kit (Agilent Technologies, CA, USA). An Agilent DNA Microarray Scanner G2505C (Agilent Technologies, CA, USA) was used for the acquisition and analysis of hybridization intensities.

For the microarray experiment, tissues from 4 randomly selected piglets per experimental group were sampled, representing extracted tooth buds from each experimental group. For the purposes of the microarray experiment, all the studied samples were hybridized against the pool of equal amounts of RNA originating from all pigs participating in the study. The cRNA of common reference was labeled with Cy3 and the cRNA obtained from investigated tissues was labeled with Cy5. Sixteen, dual-color microarrays were performed, each representing specific pigs (*n* = 4 microarrays with tooth bud samples from group 1; *n* = 4 microarrays with tooth bud samples from group 2; *n* = 4 microarrays with tooth bud samples from group 3 and *n* = 4 microarrays with tooth bud samples from group 6). Equal amounts of cRNA samples (300 ng) were hybridized on each slide. Since the microarrays were dual-color, on each slide, we hybridized one Cy3-labeled common reference sample and one Cy5-labeled experimental pig sample. Once the microarrays were scanned, after hybridization and washing, they were analyzed using the Agilent Feature Extraction (FE) software, version 10.7.3.1. FE. This software extracts the data and performs its background subtraction as well as lowness normalization of obtained intensities.

The probe intensities were then downloaded to PANTHER Version 13.0 software (Protein ANalysis THrough Evolutionary Relationships) and Pathway Studio 11.4.0.8 software (Ariadne Genomics, Rockville, MD, USA) to perform ontologic analyses and identify differentially regulated signaling pathways and functions of differentially regulated genes.

### 4.5. Morphometric and Histological Analysis of the Jawbone of Piglets

Mandibular tissues collected at euthanasia were decalcified with a 10% ethylenediaminetetraacetic acid disodium solution for 6 weeks at 4 °C and then embedded in paraffin. Serial paraffin sections were sagitally cut at a thickness of 4.5 μm using a Retratome (REM-700; Yamato Koki Industrial, Asaka, Japan). The sections were then processed for staining with standard hematoxylin-eosin. The histological structure of the bones and the osteointegration of the implanted fillers—collagen and TCP with the recipient tissues—were evaluated.

### 4.6. Statistics

The data obtained in the study were analyzed using an unpaired *t*-test, Mann–Whitney test, one-way ANOVA, and Tukey’s post hoc test or Kruskal–Wallis test (*p* < 0.05) (Prism 6 for Mac OS X, Version 6.0h, GraphPad Software, Inc., Boston, MA, USA).

## 5. Conclusions

Considering the results obtained, we believe that this is one of the first comprehensive studies on the effect of the lack of dental hinges on the growth of facial bones in a swine model. Tooth loss due to physical trauma, tooth decay, genetic defects, and aging are common health problems. Tooth agenesis is also associated with abnormal jawbone development. Although it has not been possible to identify the most effective algorithm for growth factor application to achieve normal jaw development, the lack of significant results in treatment efficacy is also a very clinically important outcome that can affect the treatment regimen and patient comfort, resulting in restrictions on the use of unnecessary surgical procedures. The developed animal model of bone development disorders (including anodontia) and the scheme for evaluating the efficacy and safety of using replacement therapy for craniofacial malformations are important in the development of the discipline and are important contributions to the introduction of treatment methods.

## Figures and Tables

**Table 1:** External manual mandibular measurements (mm) of piglets in groups 1, 2, 3, and 6; L—left side; P—right side, (mean ± SD).

	1		2		3		6	
	L	P	L	P	L	P	L	P
**1 (mm)**	9.70 ± 1.2	9.75 ± 2. 3	10.50 ± 2.5	10.5 ± 3.5	12.05 ± 2.9	12.05 ± 2.1	11.90 ± 1. 6	11.9 ± 1.6
**2**	7.10 ± 0.7	6.90 ± 3.8	8.00 ± 1.7	7.85 ± 2.1	9.40 ± 2.4	9.15 ± 3.3	8.90 ± 1.1	8.60 ± 1.5
**3**	5.60 ± 3.4	5.90 ± 2.4	5.90 ± 1.1	5.95 ± 0.9	6.70 ± 0.9	6.85 ± 1.6	6.30 ± 1.9	6.40 ± 2.0
**4**	4.50 ± 2.3	4.50 ± 1.5	4.75 ± 2.1	4.70 ± 2.1	5.20 ± 3.5	5.20 ± 2.3	5.20 ± 1.9	5.10 ± 0.9
**5**	6.90 ± 1.1	6.60 ± 2.0	7.35 ± 2.0	7.10 ± 3.4	8.60 ± 2.6	8.25 ± 1.8	7.80 ± 2.3	7.80 ± 1.7
**6**	3.00 ± 0.5	2.85 ± 1.0	3.20 ± 1.1	2.95 ± 0.9	3.45 ± 0.8	3.35 ± 1.5	3.60 ± 0.7	3.20 ± 1.9
**6′**	2.00 ± 0.7	2.00 ± 0.7	1.95 ± 0.4	1.95 ± 0.5	2.25 ± 0.2	2.30 ± 0.5	2.10 ± 1.0	2.05 ± 0.6
**7**	2.80 ± 0.5	2.40 ± 1.2	3.10 ± 0.9	2.65 ± 0.7	3.40 ± 0.9	2.90 ± 0.6	3.50 ± 0.7	2.70 ± 0.9
**7′**	1.65 ± 0.3	1.50 ± 0.5	1.75 ± 0.8	1.65 ± 0.2	2.00 ± 1.0	1.90 ± 0.5	1.90 ± 0.5	1.80 ± 1.0
**8**	3.10 ± 0.6	2.50 ± 0.6	3.20 ± 0.6	2.45 ± 0.3	3.50 ± 2.3	3.00 ± 1.0	3.60 ± 1.0	2.70 ± 0.7
**8′**	1.50 ± 0.5	1.40 ± 0.2	1.35 ± 0.5	1.35 ± 0.4	1.90 ± 0.8	1.75 ± 0.9	1.65 ± 0.6	1.65 ± 0.4
**9**	3.15 ± 1.0	2.90 ± 0.3	3.45 ± 1.0	3.35 ± 1.3	3.75 ± 0.3	3.60 ± 1.0	3.85 ± 1.4	3.60 ± 1.0
**9′**	1.35 ± 0.3	1.25 ± 0.2	1.40 ± 0.4	1.35 ± 0.5	1.85 ± 1.0	1.70 ± 0.7	1.60 ± 0.9	1.60 ± 0.8
**10**	4.30 ± 2.1		4.70 ± 1.9		5.40 ± 2.9		5.60 ± 1.7	

Abbreviations: L = left side of the mandible; P = right side of the mandible; 1 = length of mandibular molar from the first molar to the angle of the mandible; 2 = height of the mandibular branch; 3 = width of the mandibular branch at the base; 4 = width of the mandibular branch at the base of the condylar articulation; 5 = distance from the lowest point of the mandibular angle to the most anterior point of the beak process; 6–9 = height of the mandibular shaft and alveolar part; 6′–9′ = width of the alveolar part of the mandible; 10 = length of mandibular molar from the first molar to the mandibular conjunctiva.

**Table 2:** Internal radiological mandibular measurements (mm) of piglets in groups 1, 2, 3, and 6; L—left side; P—right side, (mean ± SD).

	1		2		3		6	
	L	P	L	P	L	P	L	P
**A6**	20.4 ± 3.4	20.6 ± 2.3	20.4 ± 2.4	20.6 ± 2.6	23.6 ± 3.4	24.0 ± 8.5	22.8 ± 5.2	21.6 ± 2.4
**B6**	16.2 ± 2.1	16.4 ± 0.9	15.8 ± 0.9	16.6 ± 3.4	19.0 ± 2.1	20.6 ± 1.5	18.3 ± 2.5	17.3 ± 3.5
**C6**	2.2 ± 0.7	2.4 ± 0.6	2.2 ± 0.5	2.2 ± 0.9	2.0 ± 0.7	1.4 ± 0.5	1.7 ± 0.1	1.9 ± 0.6
**D6**	2.0 ± 0.5	1.8 ± 0.7	2.6 ± 1.0	1.8 ± 2.4	2.6 ± 0.4	2.0 ± 0.6	2.8 ± 0.4	2.4 ± 0.3
**E6**	11.2 ± 2.1	11.4 ± 2.4	15.8 ± 2.4	15.0 ± 0.9	19.4 ± 2.3	19.4 ± 5.1	17.4 ± 2.4	16.6 ± 1.4
**F6**	14.0 ± 0.9	13.6 ± 1.3	13.8 ± 0.9	13.2 ± 1.6	12.8 ± 1.8	11.6 ± 3.4	13.0 ± 2.0	11.2 ± 3.0
**G6**	5.6 ± 0.6	5.8 ± 2.0	5.4 ± 2.0	6.4 ± 1.4	4.0 ± 0.7	4.4 ± 0.5	4.8 ± 1.2	5.2 ± 0.6
**H6**	30.8 ± 3.4	30.8 ± 2.5	35.0 ± 10.0	34.6 ± 2.8	36.2 ± 9.2	35.4 ± 6.7	35.2 ± 5.3	33.0 ± 11.0
**A7**	18.2 ± 2.1	16.2 ± 0.9	18.8 ± 3.2	17.6 ± 2.3	21.6 ± 4.3	21.0 ± 2.4	20.6 ± 2.7	19.0 ± 2.8
**B7**	13.8 ± 0.9	12.2 ± 3.1	15.4 ± 2.7	14.4 ± 0.9	17.6 ± 2.0	17.3 ± 2.5	17.2 ± 0.8	15.6 ± 3.4
**C7**	2.0 ± 0.5	2.0 ± 0.4	1.4 ± 0.3	1.4 ± 0.4	2.0 ± 0.3	2.0 ± 0.9	1.6 ± 0.4	1.6 ± 0.7
**D7**	2.4 ± 0.6	2.0 ± 0.2	2.0 ± 0.1	1.8 ± 0.3	2.0 ± 0.2	1.7 ± 0.6	1.8 ± 0.3	1.8 ± 0.5
**E7**	14.8 ± 3.7	16.0 ± 0.5	16.0 ± 2.1	18.0 ± 2.3	15.6 ± 0.9	19.0 ± 2.5	20.6 ± 2.0	20.2 ± 4.5
**F7**	12.2 ± 1.9	3.6 ± 1.5	13.4 ± 2.4	6.2 ± 1.7	16.8 ± 1.5	8.0 ± 1.7	11.2 ± 3.1	4.2 ± 0.6
**G7**	5.0 ± 0.5	5.4 ± 2.0	3.8 ± 0.9	4.8 ± 0.8	3.8 ± 0.5	4.0 ± 2.4	4.0 ± 0.3	4.2 ±0.3
**H7**	32.0 ± 4.6	25.0 ± 2.6	33.2 ± 1.9	29.0 ± 2.4	36.2 ± 6.4	31.0 ± 4.5	35.8 ± 9.0	28.6 ± 2.1
**A8**	16.4 ± 2.7	16.0 ± 0.6	16.8 ± 0.7	17.4 ± 1.6	20.4 ± 3.1	19.6 ± 2.9	19.0 ± 2.1	18.4 ± 2.5
**B8**	10.2 ± 1.8	11.2 ± 1.6	10.6 ± 1.4	12.0 ± 1.7	14.8 ± 2.6	14.8 ± 1.6	11.2 ± 0.9	10.6 ± 0.7
**C8**	2.2 ± 0.5	2.2 ± 0.9	1.8 ± 1.0	2.0 ± 0.7	2.0 ± 0.7	2.0 ± 0.4	2.6 ± 0.6	2.2 ± 0.6
**D8**	4.0 ± 0.9	2.6 ± 0.5	4.4 ± 0.8	4.0 ± 0.5	3.6 ± 0.6	2.8 ± 1.0	5.2 ± 2.1	5.6 ± 1.2
**E8**	10.6 ± 2.1	9.8 ± 1.4	11.4 ± 2.0	13.2 ± 0.9	15.0 ± 0.4	16.0 ± 4.5	12.4 ± 1.8	10.6 ± 3.1
**F8**	15.6 ± 0.8	10.2 ± 2.1	16.8 ± 2.6	7.6 ± 1.5	19.0 ± 1.3	10.2 ± 2.7	17.8 ± 2.5	11.0 ± 2.0
**G8**	6.0 ± 2.0	6.6 ± 0.4	5.8 ± 0.8	5.6 ± 1.3	5.2 ± 2.2	5.0 ± 1.6	6.2 ± 3.0	7.0 ± 2.3
**H8**	32.2 ± 5.3	26.6 ± 5.6	34.0 ± 4.2	26.4 ± 2.8	39.2 ± 4.4	31.2 ± 10.0	36.4 ± 6.3	28.6 ± 1.9
**A9**	16.2 ± 2.4	16.6 ± 3.5	16.8 ± 2.0	16.7 ± 1.9	19.9 ± 2.1	20.3 ± 7.4	19.2 ± 3.1	18.9 ± 2.4
**B9**	9.8 ± 1.6	10.4 ± 0.9	12.4 ± 1.8	12.0 ± 2.0	15.7 ± 3.4	15.5 ± 4.5	13.1 ± 0.9	12.7 ± 3.0
**C9**	3.0 ± 0.5	2.8 ± 0.3	1.8 ± 0.9	1.9 ± 0.7	2.0 ± 0.7	2.2 ± 0.6	1.6 ± 0.4	1.6 ± 0.6
**D9**	3.4 ± 0.9	3.4 ± 1.0	2.6 ± 1.1	2.8 ± 0.8	2.2 ± 0.3	2.6 ± 0.3	4.5 ± 1.0	4.6 ± 0.4
**E9**	10.6 ± 2.1	10.6 ± 2.0	16.0 ± 3.4	16.0 ± 0.8	17.8 ± 2.4	17.9 ± 8.1	18.2 ± 2.5	18.2 ± 2.3
**F9**	18.8 ± 1.8	18.6 ± 1.4	17.2 ± 2.8	16.8 ± 1.4	13.8 ± 1.5	13.6 ± 2.4	13.6 ± 0.9	13.4 ± 3.1
**G9**	3.4 ± 0.8	3.4 ± 0.6	2.6 ± 0.9	2.8 ± 1.0	2.2 ± 0.6	2.2 ± 0.6	5.8 ± 1.0	5.8 ± 3.4
**H9**	32.8 ± 3.7	32.6 ± 4.9	35.8 ± 13.0	35.6 ± 7.1	33.8 ± 10.0	33.7 ± 4.9	37.6 ± 5.2	37.4 ± 12.0

Abbreviations: L = left side of the mandible; P = right side of the mandible; A = width of the marrow cavity; B = thickness of the lamina cribrosa of the mandibular body from the lingual side; C = thickness of the lamina compacta of the mandibular body from the vestibular side; D = width of mandibular body; E = height of the marrow cavity; F = height of the alveolar portion of the mandible; G = thickness of the compact substance of the mandibular base; H = height of the mandibular body and alveolar part in total.

**Table 3:** PDGF concentration in piglet serum (pg/mL) on days 1, 2, and 3 after unilateral mandibular germectomy.

Group	Day 1				Day 2				Day 3			
	Before	30 min After	60 min After	*p*	Before	30 min After	60 min After	*p*	Before	30 min After	60 min After	*p*
1	134 ±39	130 ± 29	131 ± 25	*0.88*	121 ± 29	119 ± 33	131 ± 23	*0.65*	135 ± 33	129 ± 27	131 ± 31	*0.83*
2	120 ± 42	123 ± 30	121 ± 40	*0.83*	119 ± 45	115 ± 29	100 ± 34	*0.38*	101 ± 29	124 ± 46	129 ± 13	*0.46*
3	115 ± 40	119 ± 21	132 ± 34	*0.85*	123 ± 37	135 ± 19	136 ± 34	*0.46*	120 ± 60	129 ± 39	118 ± 26	*0.77*
4	129 ± 29	123 ± 43	133 ± 44	*0.54*	132 ± 55	140 ± 44	132 ± 51	*0.33*	130 ± 37	121 ± 41	121 ± 40	*0.54*
5	167 ± 42	144 ± 29	149 ± 39	*0.45*	139 ± 39	141 ± 27	129 ± 28	*0.63*	149 ± 44	138 ± 29	129 ± 19	*0.86*
6	156 ± 54	165 ± 50	159 ± 42	*0.57*	141 ± 29	137 ± 36	140 ± 32	*0.66*	130 ± 29	135 ± 19	129 ± 20	*0.76*
7	121 ± 32	134 ± 39	128 ± 29	*0.23*	130 ± 54	134 ± 33	139 ± 31	*0.78*	121 ± 41	129 ± 30	131 ± 25	*0.67*

PDGF concentration on consecutive days (1, 2, and 3) before injection (before) and at 30 min and 60 min after local injection of the test substance.

**Table 4:** BMP-2 concentration in piglet serum (pg/mL) on days 1, 2, and 3 after unilateral mandibular germectomy.

Group	Day 1				Day 2				Day 3			
	Before	30 min After	60 min After	*p*	Before	30 min After	60 min After	*p*	After	30 min After	60 min After	*p*
1	227 ± 27	247 ± 41	226 ± 25	*0.43*	214 ± 44	224 ± 53	208 ± 21	*0.79*	218 ± 22	240 ± 26	220 ± 18	*0.18*
2	201 ± 34	250 ± 36	266 ± 77	*0.33*	234 ± 32	235 ± 34	200 ± 45	*0.66*	245 ± 56	256 ± 87	234 ± 65	*0.65*
3	222 ± 29	236 ± 65	241 ± 45	*0.43*	243 ± 45	199 ± 25	222 ± 55	*0.54*	200 ± 78	222 ± 39	201 ± 48	*0.46*
4	199 ± 65	245 ± 55	267 ± 26	*0.79*	287 ± 39	256 ± 67	245 ± 67	*0.23*	245 ± 49	234 ± 56	239 ± 71	*0.37*
5	234 ± 39	267 ± 25	300 ± 55	*0.38*	255 ± 29	299 ± 78	266 ± 78	*0.45*	251 ± 67	256 ± 67	254 ± 49	*0.49*
6	274 ± 73	229 ± 59	247 ± 43	*0.43*	246 ± 39	258 ± 38	254 ± 37	*0.87*	239 ± 32	259 ± 24	275 ± 64	*0.38*
7	251 ± 21	282 ± 22	260 ± 37	*0.16*	256 ± 33	257 ± 29	294 ± 31	*0.08*	248 ± 33	252 ± 29	270 ± 37	*0.49*

BMP-2 concentration on consecutive days (1, 2, and 3) before injection (before) and at 30 min and 60 min after local injection of the test substance.

**Table 5:** Signaling pathways occurring in the tooth buds of the piglets (significantly altered).

Signaling Pathways	Number of Genes Significantly Different	(Over/Under)	*p*
Angiogenesis	8	+	*2.73 × 10^−6^*
Integrin signaling pathway	7	+	*2.09 × 10^−5^*
VEGF signaling pathway	5	+	*2.53 × 10^−5^*
RAS pathway	4	+	*4.75 × 10^−4^*
PDGF signaling pathway	4	+	*6.06 × 10^−3^*
Threonine biosynthesis	1	+	*8.82 × 10^−3^*
EGF receptor pathway	3	+	*2.39 × 10^−2^*
Heterotrimeric G-protein	3	+	*2.43 × 10^−2^*

Abbreviations: VEGF = vascular endothelial growth factor; PDGF = platelet-derived growth factor; EGF = epidermal growth factor.

**Table 6:** Biological processes occurring in the tooth buds of the piglets (significantly altered).

Biological Processes	Number of Genes Significantly Different	(Over/Under)	*p*
Phosphate metabolism	8	+	*1.13 × 10^−5^*
Basic metabolic processes	56	+	*1.96 × 10^−3^*
Transport	24	+	*3.04 × 10^−3^*
Metabolism	57	+	*4.03 × 10^−3^*
Phosphate metabolism	3	+	*5.78 × 10^−3^*
Cell–matrix adhesion	4	+	*7.30 × 10^−3^*
Protein transport	15	+	*7.73 × 10^−3^*
Intracellular protein transport	15	+	*7.73 × 10^−3^*
Oxidative phosphorylation	3	+	*8.92 × 10^−3^*
Intracellular signaling cascade	15	+	*9.15 × 10^−3^*
Cellular communication	33	+	*1.04 × 10^−2^*
Signal transduction	32	+	*1.11 × 10^−2^*
Cell adhesion	13	+	*1.16 × 10^−2^*
Protein metabolism	27	+	*1.42 × 10^−2^*
Cell-to-cell adhesion	9	+	*1.78 × 10^−2^*
Development of the nervous system	12	+	*1.94 × 10^−2^*
Transport of ions	8	+	*2.15 × 10^−2^*
Cellular processes	42	+	*2.16 × 10^−2^*
Transport of cations	7	+	*2.42 × 10^−2^*
Fat metabolism	11	+	*2.52 × 10^−2^*
Metabolism of nucleic acids	3	+	*2.57 × 10^−2^*
Embryonic development	6	+	*3.49 × 10^−2^*
Vesicular transport	10	+	*4.13 × 10^−2^*
Negative regulation of apoptosis	4	+	*4.33 × 10^−2^*

**Table 7:** Experimental groups.

Group	Description
1	Control group, without surgery (*n* = 6)
2	Unilateral mandibular germectomy with administration of BMP-2 and subsequent additional applications of BMP-2 (*n* = 6)
3	Unilateral mandibular germectomy with PDGF administration and subsequent additional PDGF applications (*n* = 6)
4	Unilateral mandibular germectomy with administration of BMP-2 without subsequent additional applications (*n* = 6)
5	Unilateral mandibular germectomy with PDGF administration without subsequent additional applications (*n* = 6)
6	Unilateral mandibular germectomy without administration of growth factors with collagen filling (*n* = 6)
7	Unilateral mandibular germectomy without administration of growth factors with TCP filling (*n* = 6)

## Data Availability

The raw data supporting the conclusions of this article will be made available by the authors upon request.
